# A population-based study of traumatic brain injury incidence and mechanisms in New Zealand: 2021–2022 compared with 2010–2011

**DOI:** 10.1016/j.lanwpc.2026.101797

**Published:** 2026-01-22

**Authors:** Kelly Jones, Alice Theadom, Nicola Starkey, Irene Zeng, Shanthi Ameratunga, Suzanne Barker-Collo, Laura Wilkinson-Meyers, Braden Te Ao, Nathan Henry, Luke A. McClean, Jennifer Chua, Leah Haumaha, Michael Kahan, Grant Christey, Natalie Hardaker, Amy Jones, Anthony Dowell, Valery Feigin, BIONIC and BIONIC2 Study Groups, Kelly Jones, Alice Theadom, Nicola Starkey, Suzanne Barker-Collo, Michael Kahan, Grant Christey, Natalie Hardaker, Amy Jones, Anthony Dowell, Valery Feigin, Laura Wilkinson-Meyers, Braden Te Ao, Shanthi Ameratunga, Irene Zeng, Jennifer Chua, Leah Haumaha, Nathan Henry, Luke A. McClean, Kay Berryman, Nina Scott, Bridgette Masters-Awatere

**Affiliations:** a National Institute for Stroke and Applied Neurosciences, School of Community and Public Health, Auckland University of Technology, Auckland, New Zealand; b Brain Health Research Institute, School of Science, Auckland University of Technology, Auckland, New Zealand; c School of Psychology, Division of Arts, Law, Psychology & Social Sciences, The University of Waikato, Hamilton, New Zealand; d Biostatistics and Epidemiology, Faculty of Health and Environmental Sciences, Auckland University of Technology, Auckland, New Zealand; e School of Population Health, Faculty of Medical & Health Sciences, The University of Auckland, Auckland, New Zealand; f Population Health Gain Group, Planning, Funding and Outcomes Directorate, Health New Zealand – Te Whatu Ora, New Zealand; g School of Psychology, Faculty of Science, The University of Auckland, Auckland, New Zealand; h TBI Rehabilitation Unit and Concussion Clinic, Waikato Occupational Services Ltd, Hamilton, New Zealand; i Te Manawa Taki (formerly Midland) Trauma Research Centre, Meade Clinical Centre, Waikato Hospital, Hamilton; Waikato Clinical School, The University of Auckland, Auckland, New Zealand; j Accident Compensation Corporation, Wellington, New Zealand; k Sports Performance Research Institute New Zealand (SPRINZ), Faculty of Health and Environmental Science, Auckland University of Technology, Auckland, New Zealand; l Māori Health, Te Whatu Ora Waikato, Health New Zealand, New Zealand; m Department of Primary Health Care and General Practice, University of Otago - Wellington, New Zealand

**Keywords:** Traumatic brain injury, Incidence, Mechanism, Population-based, Epidemiology

## Abstract

**Background:**

Monitoring traumatic brain injury (TBI) incidence and epidemiological patterns is important for evidence-based strategic planning, policy, prevention, and resource allocation. We revisited population-based estimates and examined patterns of TBI incidence (all ages, severities) in 2021–2022 compared with 2010–2011 in New Zealand (NZ).

**Methods:**

Examining an urban (Hamilton) and rural (Waikato District) region in NZ (May 2021–April 2022, unintentionally following the start of the COVID-19 pandemic), we calculated crude annual age-, sex-, ethnic-, urban/rural area- and mechanism-specific TBI incidence per 100,000 person-years with 95% Confidence Intervals (CI). Poisson regression was used to derive adjusted Risk Ratios (aRRs) to compare age-standardised rates between sex, ethnicity, and area groups. Direct standardisation was used to age-standardise rates to the world population. We calculated Incidence Rate Ratios (IRRs) with 95% CI to compare 2021–2022 with 2010–2011 age-standardised rates.

**Findings:**

Total TBI incidence per 100,000 person-years was 852 cases (95% CI 816–890), including 791 cases (756–828) of mild TBI, and 61 cases (52–72) of moderate to severe TBI. TBI affected males more than females (IRR 1.31, 95% CI 1.29–1.33), and urban more than rural residents (IRR 1.57, 1.43–1.73). Most TBI (61%) occurred in people aged 15–64 years and were due to falls (48%). European and Asian peoples had lower risk of TBI than Māori (aRRs 0.68, 0.31 respectively). Compared to 2010–2011, total TBI incidence and rates among Māori were stable; TBI incidence was greater among females, urban residents, and adults aged ≥34 years; and TBI due to falls significantly increased (IRR 1.20, 95% CI 1.03–1.40).

**Interpretation:**

Noting increased risks for underestimation due to COVID-19, findings suggest overall TBI incidence rate in NZ was similar in 2021–2022 to 2010–2011, while highlighting changes in TBI distribution. Age-, sex-, area-, ethnic-, and mechanism-specific distributions should be considered when revisiting prevention strategies to reduce TBI incidence.

**Funding:**

10.13039/501100001505Health Research Council of New Zealand of NZ.

**Research in context**

**Evidence before this study**

Prior to the first BIONIC study in 2010–2011, we searched Medline with the terms “traumatic brain injury”, “epidemiology”, “incidence”, “ethnic/racial”, AND “population or community based” for population-based studies of traumatic brain injury (TBI) published between 1960 and 2012. At this time, most studies were not population-based, limited to hospitalised TBI patients, and used varying diagnostic criteria that limited accurate population-based estimates of TBI burden. To our knowledge, no population-based study including multiple sources of case ascertainment to capture hospitalised, non-hospitalised, and fatal cases has examined age-, sex-, ethnic-, area (urban, rural) and mechanism-specific rates of TBI (including all ages, all severities), including pre- and following the start of the COVID-19 pandemic.

**Added value of this study**

Our population-based BIONIC studies extend previous evidence by assessing the incidence of all severities of TBI including both sexes, all age groups, urban and rural populations, and all injury mechanisms. We also compared TBI incidence rates overall and by age, sex, ethnicity, TBI severity and mechanism groups between two time points that uniquely examined incidence both before and during the COVID-19 pandemic. We found that the overall TBI incidence rate in NZ was similar in 2021–2022 to 2010–2011, but the distribution of TBI had changed in some groups. Findings provide important insight into the impacts of the COVID-19 pandemic on population-based patterns of TBI incidence that may reflect changes in healthcare seeking barriers and behaviour during the pandemic. Government responses to COVID-19 may have also led to changes in TBI incidence rates across different TBI mechanisms.

**Implications of all the available evidence**

Based on our study and existing evidence, significant increases in TBI incidence among people aged ≥65 years, alongside an ageing population structure, highlights an important area for prevention. Relatedly, there is a need to reduce falls more broadly—being a persistent leading cause of TBI among older and younger populations, including during pandemic conditions. Findings highlight the importance of raising TBI awareness, improving TBI recognition, and informing health service planning to respond to increases in TBI incidence among older people. Findings also highlight the need for TBI care providers and policymakers to identify ways to improve healthcare access and uptake during pandemic conditions. Our age-, sex-, area-, ethnic-, and mechanism-specific data should be considered when revisiting tailored prevention strategies and care for age, sex and area groups and to reduce TBI burden at total population and sub-population levels. Given noted changes in TBI epidemiology, on-going monitoring of TBI incidence is warranted.

## Introduction

Traumatic brain injury (TBI) is a substantial global public health burden, with significant long-term physical, social, emotional, and cognitive consequences.^1^ Economic costs include acute healthcare, disability compensation, and rehabilitation,^2^^,^^3^ globally costing ∼ US$400 billion annually.^1^ Pre-COVID-19, TBI incidence per 100,000 ranged from 349 internationally,^4^ up to 694 in Europe,^5^ and 790 in New Zealand (NZ).^6^ Since COVID-19, changes in TBI epidemiology have been reported, including increases in TBI due to violence, and decreases in TBI due to traffic accidents during lockdowns.^7^ Such changes likely reflect restrictions on mobility and activities,^8^ financial and social impacts,^9^ and barriers accessing healthcare.^10^ Updated data on TBI incidence and epidemiological patterns are required for evidence-based strategic planning, policy, prevention, and resource allocation. We aimed to revisit age-, sex-, ethnic-, area (urban, rural) and mechanism-specific rates of mild and combined moderate to severe TBI in NZ in the Brain Injury Outcomes NZ In the Community (BIONIC2; 2021–2022) study compared to rates from the first BIONIC (2010–2011) study.

## Methods

Our population-based studies, undertaken in the Hamilton (urban) and Waikato District (rural) in NZ, sought to register all TBI over two 12-month periods (BIONIC 01 March 2010, to 28 February 2011; BIONIC2 01 May 2021 to 30 April 2022).

### Study population

The BIONIC studies included people of all ages in a large geographical area in the Central North Island of NZ, spanning Hamilton City (98 km^2^) and surrounding rural area (Waikato District, 31,987 km^2^). NZ Census 2006 data (a 5-yearly snapshot of the NZ population) were used in BIONIC estimates, reporting 129,249 Hamilton City and 43,956 Waikato District residents (55% European, 20% Māori [the indigenous population of NZ], 2% Pacific Peoples, 15% of other ethnic origins), with rural (25%) to urban (75%) proportions. NZ Census 2018 data were used in BIONIC2 estimates, reporting 160,941 Hamilton City and 75,570 Waikato District residents (55% European, 32% Māori, 4% Pacific Peoples, 9% people of other ethnic origins), with rural (22%) to urban (78%) proportions. The study population was relatively representative of the national population for age, sex, socio-economic mix, ethnicity, and rural/urban proportions.^11^

### Procedures

Both studies used consistent prospective and retrospective approaches to case ascertainment. TBI was defined using World Health Organisation criteria, being ‘an acute brain injury resulting from mechanical energy to the head from external physical forces’.^12^ Operationally, TBI required one or more criteria: confusion or disorientation; loss of consciousness; post-traumatic amnesia; or other neurological abnormalities (i.e. intracranial lesion). Symptoms were related to TBI and not due to illicit drugs, alcohol, medication use, other injuries, treatments, or other problems (e.g., psychological). In unclear cases, clinical evidence was reviewed by a Diagnostic Adjudication Group, with consistent membership across studies. Using standard definitions,^13^, ^14^, ^15^ mild TBI (including concussion) was defined as a Glasgow Coma Scale (GCS) score of 13–15 and/or post-traumatic amnesia (PTA, <24 h); and the presence of ≥1 of the above operational criteria. Moderate TBI was defined as GCS 9–12 or PTA within 1–6 days of injury. Severe TBI was defined as ≤ GCS 8 or PTA ≥7 days from injury. Where available, GCS scores were recorded at the scene of TBI, admission to medical service, or both. Individuals' worst GCS score, recorded within the first 4 weeks of injury, captured changes in scores. If GCS and PTA severities differed, the more severe category was assigned. In the absence of PTA, severity was based on GCS score/s. If PTA and GCS were unavailable, TBI severity was deemed mild. Given difficulties applying criteria to children (e.g., determining confusion in infants), head injury followed by behavioural changes (e.g., persistent crying) were required to confirm TBI. Mechanism was classified using the ICD-10 external cause classification system and grouped by falls (e.g., trips), transport incidents (traffic and non-traffic related), assaults (e.g., interpersonal violence), exposure to mechanical forces (e.g., being accidentally struck by a person, animal, or inanimate object), and other (unknown or unspecified) causes. Sports-related TBI were predominantly coded as falls or exposure to mechanical force.

**Fig. 1:**
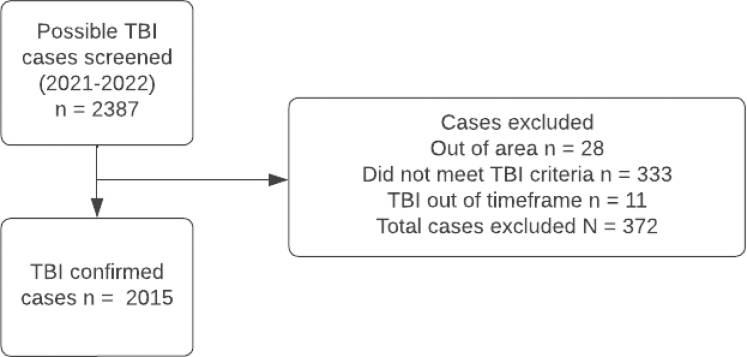
**Overview of TBI case ascertainment**.

Case ascertainment, based on multiple overlapping sources of information, included hospitalised, non-hospitalised, fatal and non-fatal TBI (Fig. 1). Before the study, all GP practices in the region were informed about the study and asked to help identify cases, with information kits provided. Of the three GP practices which declined participation, one agreed to make study brochures available to patients. Throughout the case ascertainment period, with consistent incentives across both studies, regular GP contact was via an agreed method (e.g., phone, email), alongside weekly in-person visits, periodic newsletters, and on-site meetings. One large hospital (about 600 beds) provided comprehensive acute, including neurosurgical care, for TBI patients in the study region. Daily checks included the review of all hospital and emergency department (ED) records (including trauma cases, computerised tomography/magnetic resonance imaging records, and the hospital discharge register); weekly checks included all community health services; and an overarching check of ambulance services, coroner/autopsy records, aged-care facilities, and the Accident Compensation Corporation (ACC) database. The ACC is a government-supported no-fault insurance agency that funds treatment and rehabilitation for all NZ residents with accidental injuries. Efforts to capture mild TBI cases not admitted to hospital included invited referrals of new and suspected TBI cases from family doctor practices and undertaking cross checks of accident records of schools, physiotherapy providers and sports centres (within and just outside of the study area), and through self-referrals via study advertisements. Final case ascertainment checks included reviewing computerised hospital separations data for public hospitals with ICD-10 S00–S09 codes for head injury (via National Health Index number). All TBI cases were cross checked against the study registry to identify duplicates, including lists of previously excluded cases and from other sources (i.e., smaller hospitals in the larger Waikato region, schools, sports groups, aged-care facilities, concussion services, ACC).

The following changes, approved by the study Operations Team, were made due to extenuating circumstances. On 19 May 2021, hospital record searches were halted due to a ransomware attack preventing computer access. Staff hours were reallocated to support case identification once access to electronic records resumed 4–5 weeks later and for checking manual ED records from the impacted period. During the incidence period, the study region was under lockdown or significant restrictions for approximately nine months. From August 2021, Covid-19 restrictions and related pressures on the health system disrupted weekly visits to community health providers and reduced study engagement. Mitigating strategies included phone call contact with each provider.

### Statistical analysis

Data management incorporated exploratory analyses, including examination of age and frequency distributions (e.g., histogram, QQ plots, tabulation), to detect any systematic and/or random error in the study dataset. We calculated crude annual incidence of TBI per 100,000 with 95% CIs using Wilson exact method for binomial distribution (due to low incidence in some subpopulations). Age and sex structures of the NZ population census data for 2018 for the Hamilton City (urban) and Waikato District (rural) were used as denominators. Age-standardised TBI incidence rates were calculated based on WHO world populations. Prioritised ethnic origin was grouped by 1) Māori, Pacific Peoples, Asian, Other, and European people; and 2) Māori, European and Other (combined Pacific Peoples/Asian/Other) for analyses describing the TBI incidence sample, and for calculating unadjusted and adjusted risk ratios. Age-, sex-, area- (urban, rural), and ethnic-specific (Māori, European, Other (combined Pacific Peoples/Asian/Other)) incidences were calculated for total TBI, mild and combined moderate to severe TBI, and mechanisms. Multiple Poisson regression^16^ was used to derive adjusted risk ratios (aRRs) for sex, age-group, ethnicity, and area. To account for variation in population size across strata, the logarithm of the total population count for each combination of these variables was incorporated as an offset term,^17^ thereby ensuring unbiased comparisons. Risk ratios (RRs) and incidence rate ratios (IRR) with 95% CIs (using Exact and Normal approximation for Poisson approaches) were used to compare the age-standardised rates between published BIONIC data and BIONIC2 by sex, ethnicity, area, TBI severity, and mechanism groups. To compare incidence rates for different injury mechanisms by age groups, we calculated crude incidence per 100,000 without standardisation.

#### Ethics approval

Both studies were approved by the NZ Health and Disability Ethics Committee (BIONIC NTY/09/09/095; BIONIC2 21/NTB/11).

### Role of the funding source

The funder had no role in study design, data collection, data analysis, interpretation and writing of the report. The corresponding author had full access to all study data and had final responsibility for the decision to submit for publication.

## Results

### BIONIC2 (2021–2022)

Of the 2015 individuals with TBI identified in 2021–2022, most were aged 15–64 years (61% [1231]), males (55% [1105]), Europeans (55% [1112]), urban residents (78% [1575]), with most TBI classified as being of mild severity (93% [1871], Table 1). Most moderate to severe TBI (84% [121]) and mild TBI (82% [1531]) were in those aged >15 years. Nearly half of all TBI occurred in a home/private house/grounds (43%). Most TBI were identified through the main hospital (72% [1453]), with 27% [536 of 2015] identified through non-hospital sources (e.g., ACC 16% [320], Accident and Medical Clinics 2% [35]).

**Table 1:** Characteristics of incidence sample (2021–2022).

	Mild TBI (n = 1871)	Moderate to severe TBI (n = 144)	Total TBI (n = 2015)
Mean age in years (SD)	39.3 (26.4)	38.0 (25.0)	39.2 (26.3)
Median age in years (IQR)	33 (18–67)	32 (18–57)	33 (18–60)
Male	1017 (54.36%)	88 (61.11%)	1105 (54.8%)
Urban residency	1461 (78.09%)	114 (79.17%)	1575 (78.2%)
Ethnic origin			
Māori	580 (31.0%)	60 (41.67%)	640 (31.8%)
European	1046 (55.91%)	66 (45.83%)	1112 (55.2%)
Pacific Peoples	71 (3.79%)	6 (4.17%)	77 (3.8%)
Asian	114 (6.09%)	6 (4.17%)	120 (6.0%)
Other	60 (3.21%)	6 (4.17%)	66 (3.3%)
Site of case detection by study team			
Hospital	1382 (73.86%)	71 (49.31%)	1453 (72.1%)
Family doctor	25 (1.34%)	1 (0.69%)	26 (1.3%)
Other (e.g., Accident and Medical Centre)	464 (24.8%)	72 (50.0%)	536 (26.6%)
Cause of TBI			
Transport incident	355 (18.97%)	35 (24.31%)	390 (19.3%)
Fall	904 (48.32%)	57 (39.58%)	961 (47.7%)
Exposure to mechanical force	293 (15.66%)	27 (18.75%)	320 (15.9%)
Assault	298 (15.93%)	20 (13.89%)	318 (15.8%)
Other and Unknown (not specified)	21 (1.12%)	5 (3.47%)	26 (1.3%)
Location			
Home/private house/grounds	818 (43.72%)	59 (40.97%)	877 (43.5%)
School	57 (3.05%)	6 (4.17%)	63 (3.1%)
Highway/Road/Street	383 (20.47%)	36 (25.0%)	419 (20.8%)
Recreational area	172 (9.19%)	17 (11.81%)	189 (9.4%)
Work	130 (6.95%)	5 (3.47%)	135 (6.7%)
Unknown	136 (7.27%)	6 (4.17%)	142 (7.0%)
Other	175 (9.35%)	15 (10.42%)	190 (9.4%)
Activity			
Work	118 (6.31%)	6 (4.17%)	124 (6.2%)
Travelling	264 (14.11%)	27 (18.75%)	291 (14.4%)
Leisure	352 (18.81%)	22 (15.28%)	374 (18.6%)
In a conflict	292 (15.61%)	18 (12.5%)	310 (15.4%)
Sport	140 (7.48%)	14 (9.72%)	154 (7.6%)
Unknown	184 (9.83%)	21 (14.58%)	205 (10.2%)
Other	521 (27.85%)	36 (25.0%)	557 (27.6%)

Data are n (%), unless otherwise stated. TBI = traumatic brain injury. Note: Some totals do not add up to 100% due to rounding.

Total TBI incidence was 852/100,000 person-years (age-standardised rate 826/100,000 person-years, Table 2). The risk of mild TBI was more than 13 times greater than the risk of moderate to severe TBI. Mild TBI was most common in males and females aged ≥65 years. Moderate to severe TBI was most common in men aged 15–34 years, and in women aged ≥65 years. Compared to females, age-standardised incidence in males was almost one and a third times greater for total TBI (IRR 1.31, 95% CI 1.29–1.33) and mild TBI (IRR 1.28, 95% CI 1.26–1.30), and nearly two times greater for moderate to severe TBI (IRR 1.85, 95% CI 1.61–1.99). Total age-standardised incidence of TBI was significantly higher among Māori people (1120/100,000 person-years) than European (777/100,000 person-years) and Pacific Peoples/Asian/and people of other ethnic origins combined (604/100,000 person-years, Table 3). Māori people aged 15–34 years had higher TBI incidence than people of the same age range in European and Pacific Peoples/Asian/and people of other ethnic origins combined. The IRR of age-standardised incidence between European and Māori for mild TBI was 0.72 (95% CI 0.70–0.74) and for moderate to severe TBI was 0.39 (95% CI 0.33–0.44). Between Pacific Peoples/Asian/and people of other ethnic origins combined and Māori, the IRR was 0.56 (95% CI 0.50–0.62) for mild TBI and 0.25 (95% CI 0.21–0.30) for moderate to severe TBI. Compared to rural residents, age-standardised incidence in urban residents was around one and a half times greater for mild (IRR 1.57, 95% CI 1.42–1.73) and moderate to severe TBI (IRR 1.64, 95% CI 1.16–2.15) (Table 4).

**Table 2:** Traumatic brain injury incidence by age, sex, and severity of injury (2021–2022).

	Total regional general population (n) (NZ Census 2018)	Mild TBI		Moderate to severe TBI		Total TBI	
		Population (n)	Incidence per 100,000 person-years (95% CI)	Population (n)	Incidence per 100,000 person-years (95% CI)	Population (n)	Incidence per 100,000 person-years (95% CI)
**Boys and men**							
0–4	8670	79	911 (732–1134)	7	81 (39–167)	86	992 (804–1223)
5–14	18,003	136	755 (639–893)	12	67 (38–116)	148	822 (700–965)
15–34	35,169	352	1001 (902–1110)	37	105 (76–145)	389	1106 (1002–1221)
35–64	41,469	274	661 (587–743)	20	48 (31–74)	294	709 (633–794)
65 and over	13,143	176	1339 (1156–1550)	12	91 (52–160)	188	1430 (1241–1648)
Total	116,454	1017	873 (821–928)	88	76 (61–93)	1105	949 (895–1006)
Standardised^b^	–	–	862 (754–988)	–	77 (50–122)	–	940 (826–1070)
Rate Ratio (REF: Girls and women)	–	–	1.28 (1.26–1.30)	–	1.85 (1.61–1.99)	–	1.31 (1.29–1.33)
**Girls and women**							
0–4	8292	55	663 (510–862)	a	a	a	a
5–14	17,181	70	407 (323–514)	a	a	a	a
15–34	35,115	272	775 (688–872)	18	51 (32–81)	290	826 (736–926)
35–64	44,100	240	544 (480–617)	18	41 (26–65)	258	585 (518–661)
65 and over	15,369	217	1412 (1237–1611)	16	104 (64–169)	233	1516 (1335–1722)
Total	120,057	854	711 (665–760)	56	47 (36–61)	910	758 (710–809)
Standardised^b^	–	–	674 (581–784)	–	42 (25–76)	–	716 (620–828)
**Total**							
0–4	16,962	134	790 (667–935)	7	41 (20–85)	141	831 (705–979)
5–14	35,184	206	585 (511–671)	16	45 (28–74)	222	631 (553–719)
15–34	70,284	624	888 (821–960)	55	78 (60–102)	679	966 (896–1041)
35–64	85,569	514	601 (551–655)	38	44 (32–61)	552	645 (594–701)
65 and over	28,512	393	1378 (1249–1520)	28	98 (68–142)	421	1477 (1343–1623)
Total	236,511	1871	791 (756–828)	144	61 (52–72)	2015	852 (816–890)
Standardised^b^	–	–	767 (694–849)	–	59 (42–85)	–	826 (750–911)

TBI, traumatic brain injury.

a Number of cases <5 were not presented. Total counts are not presented when numbers for one or more groups required suppression.

b Standardised to the WHO World 2000 standard population.

**Table 3:** Traumatic brain injury incidence by ethnicity origin and severity of injury (2021–2022).

	Total regional general population (n)	Mild TBI		Moderate to severe TBI		Total TBI	
		Population (n)	Incidence per 100,000 person-years (95% CI)	Population (n)	Incidence per 100,000 person-years (95% CI)	Population (n)	Incidence per 100,000 person-years (95% CI)
**European**							
0–4 years	7032	51	725 (552–952)	a		a	
5–14 years	15,960	89	558 (453–686)	a		a	
15–34 years	33,993	291	856 (764–960)	28	82 (57–119)	319	938 (841–1047)
35–64 years	52,929	286	540 (481–606)	15	28 (17–47)	301	569 (508–636)
≥65 years	23,292	329	1413 (1269–1572)	18	77 (49–122)	347	1490 (1342–1654)
Total	133,206	1046	785 (739–834)	66	50 (39–63)	1112	835 (787–885)
Standardised^b^	–	–	729 (632–843)	–	48 (30–83)	–	777 (677–894)
Rate Ratio (Ref: Māori)	–	–	0.72 (0.70–0.74)	–	0.39 (0.33–0.44)	–	0.52 (0.49–0.55)
**Māori**							
0–4 years	6387	55	861 (662–1119)	5	78 (33–183)	60	939 (731–1207)
5–14 years	12,849	84	654 (528–809)	12	93 (53–163)	96	747 (612–911)
15–34 years	19,158	244	1274 (1124–1442)	19	99 (64–155)	263	1373 (1218–1548)
35–64 years	16,860	164	973 (835–1132)	17	101 (63–161)	181	1074 (929–1241)
≥65 years	2796	33	1180 (842–1653)	7	250 (121–516)	40	1431 (1052–1942)
Total	58,050	580	999 (921–1083)	60	103 (80–133)	640	1102 (1021–1191)
Standardised^b^	–	–	1013 (854–1206)	–	107 (62–186)	–	1120 (951–1323)
**Pacific Peoples/Asian/Other**							
0–4 years	3543	28	790 (547–1140)	a		a	
5–14 years	6375	33	518 (369–726)	a		a	
15–34 years	17,133	89	519 (422–639)	8	47 (24–92)	97	566 (464–690)
35–64 years	15,780	64	406 (318–518)	6	38 (17–83)	70	444 (351–560)
≥65 years	2424	31	1279 (902–1810)	a		a	
Total	45,255	245	541 (478–613)	18	40 (25–63)	263	581 (515–656)
Standardised^b^	–	–	565 (427–752)	–	38 (16–108)	–	604 (460–795)
Rate Ratio (Ref: Māori)	–	–	0.56 (0.50–0.62)	–	0.25 (0.21–0.30)	–	–

TBI, traumatic brain injury.

a Number of cases <5 were not presented. Total counts are not presented when numbers for one or more groups required suppression.

b Standardised to the WHO World 2000 standard population.

**Table 4:** Traumatic brain injury incidence by area of residency and severity of injury (2021–2022).

	Total regional general population (n)	Mild TBI		Moderate to severe TBI		Total TBI	
		Population (n)	Incidence per 100,000 person-years	Population (n)	Incidence per 100,000 person-years	Population (n)	Incidence per 100,000 person-years
**Urban (Hamilton)**							
0–4 years	11,622	108	929 (770–1121)	7	60 (29–124)	115	990 (825–1186)
5–14 years	22,806	149	653 (557–767)	11	48 (27–86)	160	702 (601–819)
15–34 years	52,599	504	958 (878–1045)	44	84 (62–112)	548	1042 (959–1132)
35–64 years	54,915	377	687 (621–759)	29	53 (37–76)	406	739 (671–815)
≥65 years	18,999	323	1700 (1526–1894)	23	121 (81–182)	346	1821 (1641–2021)
Total	160,941	1461	908 (863–955)	114	71 (59–85)	1575	979 (932–1028)
Standardised^b^	–	–	866 (772–972)	–	68 (46–102)	–	933 (836–1043)
Rate Ratio (REF: Waikato)	–	–	1.57 (1.42–1.73)		1.64 (1.16–2.15)	–	1.57 (1.43–1.73)
**Rural (Waikato)**							
0–4 years	5340	26	487 (332–712)	a	a	a	a
5–14 years	12,378	57	460 (356–596)	5	40 (17–95)	62	501 (391–642)
15–34 years	17,685	120	679 (568–811)	11	62 (35–111)	131	741 (625–878)
35–64 years	30,654	137	447 (378–528)	9	29 (15–56)	146	476 (405–560)
≥65 years	9513	70	736 (583–929)	5	53 (22–123)	75	788 (629–987)
Total	75,570	410	543 (493–597)	30	40 (28–57)	440	582 (530–639)
Standardised^b^	–	–	552 (447–683)	–	41 (21–89)	–	593 (484–728)

TBI, traumatic brain injury.

a Number of cases <5 were not presented. Total counts are not presented when numbers for one or more groups required suppression.

b Standardised to the WHO World 2000 standard population.

Falls were the leading mechanism (48% [961]), followed by transport incidents (19% [390]), exposure to mechanical force (16% [320]), assaults (16% [318]), and other/unknown causes (1% [26]) (Table 1). Most falls occurred in people aged ≥65 years (39% [371 of 961]). Females in all age bands from 5 years and above had a greater risk of TBI from falls than males in the same age range (Table 5). Urban residents had higher TBI incidence due to falls (432 vs 235), exposure to mechanical force (161 vs 104) and assaults (155 vs 86) than rural residents. Among people aged 15–34 years, the leading TBI mechanism was transport incidents (26% [180 of 679]), followed by assaults (26% [177]). Males in age bands from 0 to 64 years had greater risk of TBI from transport incidents than females in the same age groups. TBI due to assaults was more common among people aged 15–34 years than any other age groups, including among males and females, urban and rural areas, with age-standardised rates higher among males than females (174/100,000 vs 97/100,000). Risk of TBI adjusted by age, sex, ethnicity, and area revealed that children (0–4, 5–14 years) had significantly lower risk of TBI with aRRs of 0.50 and 0.38 compared to people aged ≥65 years (Appendix 1). Younger (15–34 years) and older (35–64 years) adults also had lower risk of TBI with aRRs of 0.60 and 0.43 compared to people aged ≥65 years. Females had significantly lower risk of TBI than males (aRR 0.78, 0.71–0.85). European and Asian peoples had significantly lower risk of TBI than Māori with aRRs of 0.68 (0.62–0.76) and 0.31 (0.26–0.38). Pacific Peoples and people of other ethnic origins had similar risks of TBI compared to Māori with aRRs of 0.81 (0.64–1.03) and 1.0 (0.77–1.29). Urban residents had a significantly higher risk of TBI than rural residents (aRR 1.72, 1.54–1.91).

**Table 5:** Traumatic brain injury incidence by mechanism, age, sex, and area of residency (2021–2022).

	Transport incident		Fall		Exposure to mechanical force		Assault		Other or unknown	
	Number	Incidence per 100,000 person-years (95% CI)	Number	Incidence per 100,000 person-years (95% CI)	Number	Incidence per 100,000 person-years (95% CI)	Number	Incidence per 100,000 person-years (95% CI)	Number	Incidence per 100,000 person-years (95% CI)
**Boys and men**										
0–4 years	13	150 (88–256)	61	704 (548–903)	9	104 (55–197)	a	a	a	a
5–14 years	32	178 (126–251)	73	405 (323–509)	36	200 (144–277)	7	39 (19–80)	a	a
15–34 years	102	290 (239–352)	67	191 (150–242)	104	296 (244–358)	109	310 (257–374)	a	a
35–64 years	80	193 (155–240)	90	217 (177–267)	40	96 (71–131)	80	193 (155–240)	a	a
≥65 years	20	152 (99–235)	153	1164 (994–1362)	8	61 (31–120)	6	46 (21–100)	a	a
Total	247	212 (187–240)	444	381 (347–418)	197	169 (147–194)	203	174 (152–200)	14	12 (7–20)
Standardised^b^	–	215 (167–281)	–	360 (291–447)	–	183 (140–243)	–	174 (138–226)	–	12 (5–37)
**Girls and women**										
0–4 years	a	a	48	579 (437–767)	7	84 (41–174)	a	a	a	a
5–14 years	13	76 (44–129)	43	250 (186–337)	12	70 (40–122)	a	a	a	a
15–34 years	78	222 (178–277)	84	239 (193–296)	57	162 (125–210)	68	194 (153–245)	a	a
35–64 years	46	104 (78–139)	124	281 (236–335)	41	93 (69–126)	41	93 (69–126)	6	14 (6–30)
≥65 years	6	39 (18–85)	218	1418 (1243–1618)	6	39 (18–85)	a	a	a	a
Total	143	119 (101–140)	517	431 (395–469)	123	102 (86–122)	115	96 (80–115)	12	10 (6–17)
Standardised^b^	–	123 (92–170)	–	380 (311–466)	–	183 (75–106)	–	97 (73–139)	–	10 (4–33)
**Total**										
0–4 years	13	77 (45–131)	109	643 (533–775)	16	94 (58–153)	a	a	a	a
5–14 years	45	128 (96–171)	116	330 (275–395)	48	136 (103–181)	a	a	a	a
15–34 years	180	256 (221–296)	151	215 (183–252)	161	229 (196–267)	177	252 (217–292)	10	14 (8–26)
35–64 years	126	147 (124–175)	214	250 (219–286)	81	95 (76–118)	121	141 (118–169)	6	7 (3–15)
≥65 years	26	91 (62–134)	371	1301 (1176–1439)	14	49 (29–82)	6	21 (10–46)	a	a
Total	390	165 (149–182)	961	406 (381–433)	320	135 (121–151)	318	134 (120–150)	26	11 (8–16)
Standardised^b^	–	169 (139–208)	–	366 (316–425)	–	145 (117–182)	–	128 (108–151)	–	8 (4–15)
**Urban (Hamilton)**										
0–4 years	8	69 (35–136)	91	783 (638–960)	13	112 (65–191)	a	a	a	a
5–14 years	28	123 (85–177)	88	386 (313–475)	34	149 (107–208)	8	35 (18–69)	a	a
15–34 years	135	257 (217–304)	117	222 (186–266)	137	260 (220–308)	150	285 (243–335)	9	17 (9–33)
35–64 years	87	158 (128–195)	168	306 (263–356)	52	95 (72–124)	91	166 (135–203)	8	15 (7–29)
≥65 years	21	111 (72–169)	306	1611 (1441–1800)	11	58 (32–104)	6	32 (14–69)	a	a
Total	279	173 (154–195)	770	478 (446–513)	247	153 (136–174)	256	159 (141–180)	23	14 (10–21)
Standardised^b^	–	172 (136–220)	–	432 (366–510)	–	161 (126–208)	–	155 (126–196)	–	14 (6–35)
**Rural (Waikato)**										
0–4 years	5	94 (40–219)	18	337 (213–532)	a	a	a	a	a	a
5–14 years	17	137 (86–220)	28	226 (157–327)	14	113 (67–190)	a	a	a	a
15–34 years	45	254 (190–340)	34	192 (138–269)	24	136 (91–202)	27	153 (105–222)	a	a
35–64 years	39	127 (93–174)	46	150 (113–200)	29	95 (66–136)	30	98 (69–140)	a	a
≥65 years	5	53 (22–123)	65	683 (536–870)	a	a	a	a	a	a
Total	111	147 (122–177)	191	253 (219–291)	73	97 (77–121)	62	82 (64–105)	a	a
Standardised^b^	–	164 (114–240)	–	235 (169–328)	–	104 (66–169)	–	86 (57–142)	–	4 (1–34)

TBI, traumatic brain injury.

a Number of cases <5 were not presented. Total counts are not presented when numbers for one or more groups required suppression.

b Standardised to the WHO World 2000 standard population.

### BIONIC2 (2021–2022) compared to BIONIC (2010–2011)

**Fig. 2:**
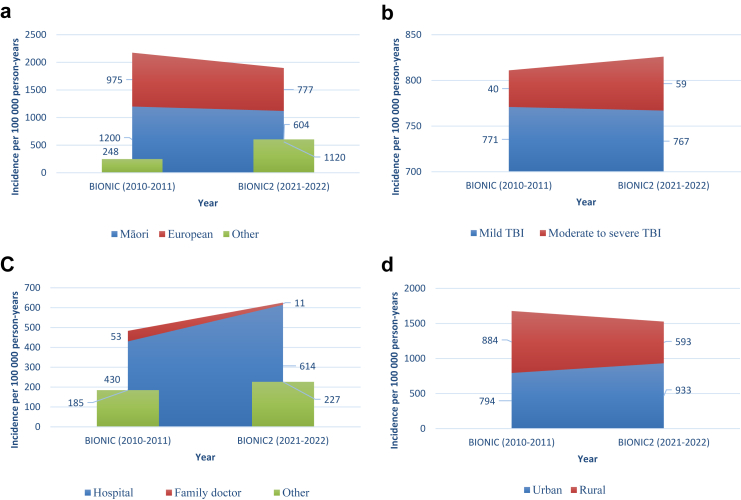
**Age-standardised TBI Incidence by ethnicity (a), severity (b), case site detection (c), and area (d) groups (2010–2011 compared with 2021–2022)**.

Compared with TBI incidence data from 2010 to 2011 (derived from the BIONIC sample, Appendix 2), there were no significant changes in the WHO age-standardised TBI incidence in 2021–2022 for total TBI (IRR 1.02, 95% CI 0.92–1.12), mild TBI (IRR 0.99, 95% CI 0.90–1.10) or moderate-severe TBI (IRR 1.48, 95% CI 0.97–2.26) (Fig. 2). In terms of age, significantly more TBI (crude incidence) were identified among people aged 35–64 years (IRR 1.27, 95% CI 1.13–1.43) from 508 (452–564) in 2010–2011 to 645 (594–701) per 100,000 in 2021–2022, and among people aged ≥65 years (IRR 2.37, 95% CI 2.16–2.60), from 623 (506–740) in 2010–2011 to 1477 (1343–1623) per 100,000 in 2021–2022. Significantly fewer TBI (crude incidence) were identified among children aged 0–4 years (IRR 0.64, 95% CI 0.59–0.70), from 1300 (1105–1496) in 2010–2011 to 831 (705–979) per 100,000 in 2021–2022, and children aged 5–14 years (IRR 0.77, 95% CI 0.70–0.86), from 818 (709–928) in 2010–2011 to 631 (553–719) per 100,000 in 2021–2022.

Significantly more TBI were identified among females (IRR 1.21, 95% CI 1.09–1.35) from 589 (539–642) in 2010–2011 to 716 (620–828) in 2021–2022, with fewer TBI identified among males (IRR 0.90, 95% 0.83–0.99) from 1041 (973–1113) in 2010–2011 to 940 (826–1070) in 2021–2022. In terms of ethnicity, significantly more TBI were identified among ‘Other’ ethnicities (including Pacific Peoples, Asian and any other minority ethnicities combined) (IRR 2.44, 95% CI 2.10–2.82), from 248 (205–301) to 604 (460–795). Significantly fewer TBI were identified among Europeans (IRR 0.80, 95% CI 0.73–0.88) from 975 (909–1046) in 2010–2011 to 777 (677–894) in 2021–2022. There were no significant changes in TBI incidence among Māori (IRR 0.93, 95% CI 0.86–1.01), being 1200 (1085–1327) in 2010–2011 and 1120 (951–1323) in 2021–2022. Regarding area, significantly more TBI were recorded among urban residents (IRR 1.18, 95% CI 1.12–1.24) from 794 (746–844) to 933 (836–1043), with fewer TBI recorded among rural residents (IRR 0.67, 95% CI 0.60–0.74) from 884 (796–983) to 593 (484–728).

In terms of mechanism, TBI incidence due to falls increased significantly (IRR 1.20, 95% CI 1.03–1.40) from 305 (280–333) to 366 (316–425), particularly among men and women aged ≥65 years. TBI incidence due to mechanical force were not significantly different between BIONIC and BIONIC2 (IRR 0.83 (95% CI 0.67–1.04), although rates significantly increased among men aged ≥65 years (IRR 4.69, 95% CI 2.58–8.4) based on crude incidence rates. TBI incidence due to transport incidents was not significantly different between BIONIC and BIONIC2 (IRR 1.04, 95% CI 0.84–1.29), although crude incidence rates significantly increased among men aged ≥65 years (IRR 3.90, 95% CI 2.74–5.54). No significant changes in TBI incidence were observed due to assaults (IRR 0.95, 95% CI 0.75–1.21), although crude incidence rates significantly increased among females aged 15–34 years (IRR 1.34, 95% CI 1.08–1.66), and males and females aged 35–64 years (males IRR 1.56, 95% CI 1.24–1.95; females IRR 1.60, 95% CI 1.16–2.23).

## Discussion

This population-based study aimed to report TBI incidence estimates for 2021–2022 and compared incidence rates with 2010–2011 in NZ, with several novel findings. Accounting for population growth and ageing, the total age-standardised TBI incidence rate per 100,000 people in NZ in 2021–2022 was similar to the rate observed in 2010–2011. Using consistent methodology, relatively stable TBI incidence rates over similar time periods have been reported for Europe^5^ and Brazil.^18^ In NZ, a 7% average annual increase in TBI-related hospitalisations was reported from 2012 to 2019.^19^ However, this retrospective record review was not population-based and used a different TBI definition to our BIONIC studies. While no single geographical area is representative of all areas, the study population was relatively representative of the national population. Extrapolating from the BIONIC2 total TBI incidence rate of 852 (816–890) per 100,000, we estimate at least 40,041 people sustain a TBI annually in NZ (based on population counts of 2018 census).

While we sought consistent case ascertainment methods across both BIONIC studies, impacts of COVID-19 on primary care and healthcare seeking behaviour likely increased risks for underestimating TBI incidence in BIONIC2. Government responses to COVID-19 may have also led to important changes in incidence across different TBI mechanisms. Nevertheless, differences in age-, sex- and area-specific TBI incidence have implications for tailored approaches to prevention and care. In BIONIC2, significantly more TBI were identified among people aged ≥65 years than any other age groups, particularly among females and mostly due to falls as reported elsewhere.^20^ Sarcopenia, osteoporosis, vision and hearing loss, medication effects, multimorbidity, and frailty increase risk of falls among older people,^21^ who are also more likely to work past retirement age and engage in more physically active lifestyles (e.g., sports and recreational activities) than previous generations. Managing TBI in older patients is further complicated by comorbidities (e.g., diabetes, cardiovascular disease), age-related changes to the brain (e.g., cerebral atrophy, increased subdural space), and anticoagulant and antiplatelet medications that enhance the risk of intracranial haemorrhages. Alongside health benefits coupled with increasing life expectancy, low-risk physical activity must be carefully managed among older adults with 22% of the world's population expected to be aged >60 years by 2050.^22^ Avoiding polypharmacy and delivering public health programs to reduce fall hazards, and promote nutrition and body balance are likely key ingredients to responding to calls for more population-based TBI prevention programs targeting older persons.^23^ Population-based impacts of a nationwide strength and balance exercise programme for people ≥65 years, introduced in NZ around the time of BIONIC2, may become evident in future TBI incidence studies.

Consistent with prior reports,^24^ we identified fewer TBI among children during the COVID-19 pandemic which likely reflects restrictions in leisure and sporting activities, and caregiver reluctance to visit health providers due to fear of contagion of COVID-19.^10^ Our findings suggest that TBI incidence may be increasing among females and raises concerns given females face greater risks of delayed recovery from TBI,^25^ potentially due to sex differences in hormone profiles,^26^ preinjury risk factors (e.g., psychiatric disorders),^27^ and psychosocial stress.^26^ Consistent with prior reports,^28^ we identified more TBI among urban than rural residents that may reflect reluctance to travel to medical care during the pandemic and disparities in TBI care in rural areas. Our findings of higher rates of TBI among females, urban residents, and adults aged ≥65 years may reflect increased awareness of the importance of healthcare following TBI, and improvements in recognition and reporting of TBI in these groups.

Many aspects of TBI incidence were similar across both BIONIC studies, with most TBI being mild in severity, greater risk among males than females, and falls being the leading TBI mechanism. Concerningly, no significant reductions in age standardised TBI incidence were observed among Māori. This finding aligns with a recent 10-year data review suggesting declines in traumatic injury hospitalisations among all ethnic groups (Asian, Pacific, and Other) in NZ, except for Māori.^29^ This persistent disparity reflects historical and ongoing socio-economic inequities—including lower income, inadequate housing, and limited access to education and healthcare—exacerbated by systemic discrimination and colonisation.^30^ Māori experience a disproportion injury burden yet have reduced access to publicly funded injury prevention services compared with non-Māori.^31^ Elevated risk factors for injury, including TBI, such as higher rates of interpersonal violence, self-harm,^32^ and chronic multi-morbidity,^33^ further underscore the need for culturally tailored interventions. Current government initiatives incorporate Kaupapa Māori-based (Māori-led) approaches grounded in mātauranga Māori (Māori knowledge) and tikanga (Māori culture), including programs such as ‘Taurite Tū’ (strength and balance program for older adults), ‘Manini Tua’ (violence prevention strategy), and iwi (tribe)-led models such as ‘Whaioranga te Pā Harakeke’, which include paeārahi (health navigators) to strengthen Māori-led care.^31^ While these initiatives represent important progress, further research is required to design, implement, and evaluate TBI prevention efforts that are co-developed with and for indigenous communities.

While we found no significant reduction in TBI due to assaults, there were significant increases among women aged 15–34 years and 35–64 years that may reflect the financial and social impacts of the COVID-19 pandemic, including increased violence against women.^9^ Importantly, a lack of access to police nor women's refuge data in our BIONIC studies may have led to underestimates of TBI due to assault. We found no significant reductions in TBI due to transport incidents, despite traffic volumes in NZ reducing by 74% following the start of COVID-19.^34^ Similarly, trauma admission rates due to transport incidents temporarily reduced by only 20–33% in the study region,^35^ suggesting high risk behaviours of road users during lockdown, before rebounding in late 2020.^36^

This finding contrasts with significant reductions in transport-related TBI in India following the start of COVID-19.^37^ However, this retrospective study examined hospital data from an undefined catchment area, with authors acknowledging that patients who perceived their injuries to be minor may have avoided a visit to ED. Given evidence of increases in excessive speed during the pandemic, with reduced traffic volumes and empty roads,^38^ our finding highlights the importance of enforcing speed restrictions during pandemic conditions.

Our study had several strengths: the population-based design, consistent definitions, and comprehensive data capture (including all ages, TBI severities, ethnic, urban and rural populations, injury mechanisms, and hospitalised and non-hospitalised TBI) at two timepoints (including before and during COVID-19) in a well-defined geographic region. Study limitations included risks for underestimating TBI incidence due to predominant reliance on people seeking medical care. There was a notable decrease in case detection by family doctors from 8% of all cases in BIONIC to 1% in BIONIC2. This decrease was likely due to increased pressures on and barriers to accessing primary care and reductions in people movement due to COVID-19. Being an incidence study, the study was not powered for particular minority group analyses. We acknowledge that small numbers in some sub-groups of the population limited the ability to make sub-group TBI incidence estimates. Further, 2018 Census data, used in BIONIC2, had a high level of non-response, especially among Māori and Pacific Peoples.

In summary, total TBI incidence rates in NZ in 2021–2022 were similar to rates in 2010–2011 but there were some differences by age, sex and area of residence. More TBI were identified among people aged ≥65 years, females and urban residents, with a notable lack of change in TBI incidence among Māori. Ongoing population-based monitoring will determine trends over time, especially within the context of changing population structures. Findings should inform strategic planning and policy, including targeted TBI awareness, recognition and prevention strategies to effectively reduce TBI incidence.

## Contributors

KJ, AT, NS, SA, LW-M, BTA, SB-C, AJ, AD and VF contributed significantly to the design of the BIONIC and/or BIONIC2 study. KJ, AT, NS, SA, LW-M, BTA and VF obtained funding for BIONIC2, and informed data analyses. KJ, AT, NS, SA, LW-M, BTA, SB-C, MK, GC, and VF participated in the supervision of the study. JC, LH and KJ supervised the day-to-day running of the study. KJ, VF, SB-C, AT, MK (BIONIC), and NS assessed diagnosis of TBI events. MK facilitated cross-checking processes with Waikato Concussion services. IZ and KJ directly accessed and verified the underlying data. IZ, with support from NH and LM, was responsible for finalising the study analysis plan, undertaking BIONIC2 incidence analyses, TBI incidence comparisons, and statistical interpretation of findings. KJ wrote the first draft of the paper. All authors contributed to discussions, interpretation of findings, and critical revision of the paper for important intellectual content, accuracy, and honesty.

## Data sharing statement

Due to local ethics requirements, we cannot share study data for public use. However, more information about the detailed study protocol and statistical analysis plan can be obtained via email from the corresponding author. Findings of incidence of TBI and mechanisms in BIONIC are described previously.^6^

## Declaration of interests

There are no conflicts of interest to declare.

## Appendix A Supplementary data

Appendix 2

Collab authors
