# Supplementary material for: A population-based study of traumatic brain injury incidence and mechanisms in New Zealand: 2021–2022 compared with 2010–2011
Source: Lancet Reg Health West Pac. 2026 Jan 22;67:101797. doi: 10.1016/j.lanwpc.2026.101797 (PMC12861184; doi:10.1016/j.lanwpc.2026.101797)
Supplement: Appendix 1 [file mmc1.docx]

**Appendix 1. Unadjusted and adjusted risk ratios of groups using Poisson regression based on Census 2018 population.**

|  |  | **Unadjusted Risk ratio (univariate regression)** | **95% Confidence interval** | | **p value** | **Adjusted Risk ratio (multi-variable regression)** | **95% Confidence interval** | | **p value** |
| --- | --- | --- | --- | --- | --- | --- | --- | --- | --- |
| **Age group** | 0–4 | 0·56 | 0·47 | 0·68 | <·0001 | 0·50 | 0·41 | 0·61 | <·0001 |
|  | 5–14 | 0·43 | 0·36 | 0·50 | <·0001 | 0·38 | 0·32 | 0·45 | <·0001 |
|  | 15–34 | 0·65 | 0·57 | 0·73 | <·0001 | 0·60 | 0·53 | 0·68 | <·0001 |
|  | 35–64 | 0·44 | 0·38 | 0·49 | <·0001 | 0·43 | 0·38 | 0·49 | <·0001 |
|  | Ref: 65 years and over | |  |  |  |  |  |  |  |
| **Sex** | Female | 0·79 | 0·73 | 0·86 | <·0001 | 0·78 | 0·71 | 0·85 | <·0001 |
|  | Ref: Male |  |  |  |  |  |  |  |  |
| **Ethnicity** | Asian | 0·37 | 0·30 | 0·45 | <·0001 | 0·31 | 0·26 | 0·38 | <·0001 |
|  | European | 0·76 | 0·69 | 0·83 | <·0001 | 0·68 | 0·62 | 0·76 | <·0001 |
|  | Other | 1·10 | 0·85 | 1·41 | 0·4818 | 1·00 | 0·77 | 1·29 | >0·90 |
|  | Pacific Peoples | 0·91 | 0·72 | 1·15 | 0·4235 | 0·81 | 0·64 | 1·03 | 0·09 |
|  | Ref: Māori |  |  |  |  |  |  |  |  |
| **Ethnicity (combined)** | European | 0·76 | 0·69 | 0·83 | <·0001 | 0·69 | 0·62 | 0·76 | <·0001 |
|  | Pacific Peoples/Asian/Other | 0·53 | 0·46 | 0·61 | <·0001 | 0·47 | 0·41 | 0·54 | <·0001 |
|  | Ref: Māori |  |  |  |  |  |  |  |  |
| **Area** | Hamilton | 1·63 | 1·06 | 1·46 | <·0001 | 1·72 | 1·54 | 1·91 | <·0001 |
|  | Ref: Waikato |  |  |  |  |  |  |  |  |

Note: Adjusted risk ratios include adjustment for the following variables: age, sex, ethnicity, and area of residence.
