# Supplementary material for: A population-based study of traumatic brain injury incidence and mechanisms in New Zealand: 2021–2022 compared with 2010–2011
Source: Lancet Reg Health West Pac. 2026 Jan 22;67:101797. doi: 10.1016/j.lanwpc.2026.101797 (PMC12861184; doi:10.1016/j.lanwpc.2026.101797)
Supplement: Appendix 2 [file mmc2.docx]

**Appendix 2. Characteristics of traumatic brain injury incidence samples in BIONIC (2010-2011) and BIONIC2 (2021-2022)**

|  | **Mild TBI** | | **Moderate to severe TBI** | | **Total TBI** | |
| --- | --- | --- | --- | --- | --- | --- |
|  | 2010 - 2011 | 2021 - 2022 | 2010 -2011 | 2021 - 2022 | 2010 - 2011 | 2021 - 2022 |
|  | n (%) | n (%) | n (%) | n (%) | n (%) | n (%) |
| Total n | 1298 | 1871 | 71 | 144 | 1369 | 2015 |
| Mean age in years (SD) | 27·5 (21·3) | 39·3 (26·4) | 38·7 (25·7) | 38·0 (25.0) | 28·1 (21·7) | 39·2 (26·3) |
| Male | 805 (62%) | 1017 (54%) | 51 (72%) | 88 (61%) | 856 (63%) | 1105 (55%) |
| Urban residency | 965 (74%) | 1461 (78%) | 43 (61%) | 114 (79%) | 1008 (74%) | 1575 (78%) |
| Ethnicity |  |  |  |  |  |  |
| Māori | 404 (31%) | 580 (31%) | 21 (30%) | 60 (41·6%) | 425 (31%) | 640 (31.8%) |
| European | 791 (61%) | 1046 (55·9%) | 46 (65%) | 66 (45%) | 837 (61%) | 1112 (55%) |
| Pacific Peoples | 40 (4%) | 71 (3·7%) | * | 6 (4%) | 51 (4%) | 77 (3.8%) |
| Asian | 42 (3%) | 114 (6%) | * | 6 (4%) | 43 (3%) | 120 (6%) |
| Other | 12 (1%) | 60 (3%) | * | 6 (4%) | 13 (1%) | 66 (3%) |
| Site of case detection |  |  |  |  |  |  |
| Hospital | 826 (64%) | 1382 (73·8%) | 56 (79%) | 71 (49%) | 882 (64%) | 1453 (72%) |
| Family doctor | 108 (8%) | 25 (1%) | * | 1 (0·69%) | 108 (8%) | 26 (1%) |
| Other | 364 (28%) | 464 (24·8%) | 15 (21%) | 72 (50%) | 379 (28%) | 536 (26·6%) |
| TBI mechanism |  |  |  |  |  |  |
| Transport incident | 249 (19%) | 355 (18.9%) | 28 (39%) | 35 (24%) | 277 (20%) | 39 (19%) |
| Fall | 488 (38%) | 904 (48%) | 28 (39%) | 57 (39·58%) | 516 (38%) | 961 (47.7%) |
| Exposure to mechanical force | 286 (22%) | 293 (15·6%) | * | 27 (18·7%) | 288 (21%) | 320 (15.9%) |
| Assault | 218 (17%) | 298 (15·9%) | 10 (14%) | 20 (13·8%) | 228 (17%) | 318 (15.8%) |
| Other/Unknown (not specified) | 57 (4%) | 21 (1%) | * | 5 (3%) | 60 (4%) | 26 (1%) |

BIONIC indicates Brain Injury Incidence and Outcomes New Zealand in the Community study. TBI = traumatic brain injury. *Number of cases < 5 were not presented.
