# Supplementary material for: A population-based study of traumatic brain injury incidence and mechanisms in New Zealand: 2021–2022 compared with 2010–2011
Source: Lancet Reg Health West Pac. 2026 Jan 22;67:101797. doi: 10.1016/j.lanwpc.2026.101797 (PMC12861184; doi:10.1016/j.lanwpc.2026.101797)
Supplement: Collab authors [file mmc3.docx]

| BIONIC and/or BIONIC2 Study Group members | |
| --- | --- |
| First names | Surnames |
| Kelly | Jones |
| Alice | Theadom |
| Nicola | Starkey |
| Suzanne | Barker-Collo |
| Michael | Kahan |
| Grant | Christey |
| Natalie | Hardaker |
| Amy | Jones |
| Anthony | Dowell |
| Valery | Feigin |
| Laura | Wilkinson-Meyers |
| Braden | Te Ao |
| Shanthi | Ameratunga |
| Irene | Zeng |
| Jennifer | Chua |
| Leah | Haumaha |
| Nathan | Henry |
| Luke A. | McClean |
| Kay | Berryman |
| Nina | Scott |
| Bridgette | Masters-Awatere |
